# Acute Small Bowel Obstruction Induced by Tirzepatide (Mounjaro)

**DOI:** 10.7759/cureus.110692

**Published:** 2026-06-11

**Authors:** Pragya Bhandari, Anuva Ray, Prashant Bhatt, Suhail Sapkota, Shreebridhi Pande, Sadia Mustarin, Nirmal Guragai, Khalid Aziz

**Affiliations:** 1 Internal Medicine, Cape Fear Valley Medical Center, Fayetteville, USA; 2 Internal Medicine, University of Michigan Health Sparrow, Lansing, USA; 3 Cardiology, Cape Fear Valley Medical Center, Fayetteville, USA; 4 Endocrinology, Diabetes and Metabolism, Southern Regional Area Health Education Center, Fayetteville, USA

**Keywords:** acute small bowel obstruction, comorbid obesity, glucose-dependent insulinotropic polypeptide (gip)/glucagon-like peptide-1 (glp-1) receptor agonist, tirzepatide or mounjaro, type 2 diabetes mellites

## Abstract

Tirzepatide (Mounjaro), a dual glucose-dependent insulinotropic polypeptide (GIP) and glucagon-like peptide-1 (GLP-1) receptor agonist, has gained widespread adoption for the management of type 2 diabetes mellitus and obesity owing to its robust efficacy in glycemic control and weight reduction. While gastrointestinal adverse effects, including nausea, vomiting, constipation, and delayed gastric emptying, are well recognized, acute small bowel obstruction represents a rare and poorly characterized complication.

We report a case of a 50-year-old woman with insulin-dependent type 2 diabetes mellitus, diabetic neuropathy, and obesity who developed acute small bowel obstruction following three months of tirzepatide therapy titrated to 12.5 mg weekly. She presented with severe abdominal pain, vomiting, abdominal distension, and syncope. Imaging confirmed small bowel obstruction without evidence of perforation, ischemia, mass lesion, or an identifiable mechanical transition point. No alternative reversible etiology for bowel obstruction was identified. Conservative management with nasogastric decompression, bowel rest, and intravenous fluid resuscitation resulted in rapid clinical improvement. Tirzepatide was discontinued, and no recurrence was observed over two months of follow-up. This case underscores small bowel obstruction as a rare but potentially serious adverse effect of tirzepatide, likely mediated by impaired gastrointestinal motility inherent to incretin-based therapy. As tirzepatide utilization continues to expand, clinicians should maintain heightened vigilance for severe gastrointestinal complications, particularly in patients with preexisting risk factors for dysmotility.

## Introduction

Tirzepatide (Mounjaro; Indianapolis, IN: Eli Lilly and Company) is a first-in-class dual agonist of the glucose-dependent insulinotropic polypeptide (GIP) and glucagon-like peptide-1 (GLP-1) receptors approved for the treatment of type 2 diabetes mellitus (T2DM) and obesity. Its potent effects on glycemic control and weight reduction have led to rapidly increasing clinical use worldwide [1,2]. Clinical trials have demonstrated greater reductions in hemoglobin A1c and body weight than several existing antidiabetic therapies, including semaglutide and insulin analogs [1-3].

Gastrointestinal adverse effects are among the most commonly reported complications of tirzepatide therapy. These include nausea, vomiting, diarrhea, constipation, abdominal discomfort, decreased appetite, gastroesophageal reflux disease (GERD), flatulence, abdominal distention, delayed gastric emptying, and, in rare cases, ileus [3]. These effects are largely attributable to incretin-mediated modulation of gastrointestinal motility and appetite regulation. GLP-1 receptor agonists reduce gastric emptying and suppress intestinal peristalsis, mechanisms that contribute to improved satiety and postprandial glycemic control but may also predispose susceptible individuals to gastrointestinal dysmotility [4,5].

Although gastroparesis and ileus have increasingly been recognized with GLP-1 receptor agonists, acute small bowel obstruction associated with tirzepatide remains rarely reported in the literature. In 2023, the U.S. Food and Drug Administration (FDA) updated labeling for semaglutide-containing medications to include ileus as a postmarketing adverse event, raising concerns regarding severe gastrointestinal motility disorders within this drug class. We report a case of acute small bowel obstruction occurring in a patient receiving high-dose tirzepatide therapy and discuss the potential pathophysiological mechanisms and clinical implications associated with this increasingly prescribed medication.

## Case presentation

A 50-year-old woman with a history of poorly controlled insulin-dependent type 2 diabetes mellitus (T2DM), diabetic peripheral neuropathy, and obesity presented to the emergency department with acute-onset diffuse abdominal pain, multiple episodes of non-bloody, non-bilious emesis, and a preceding syncopal episode.

The patient had been receiving tirzepatide for approximately three months for the management of obesity and uncontrolled T2DM. Notably, she had previously trialed semaglutide (Ozempic) and dulaglutide (Trulicity), both of which were discontinued due to severe gastrointestinal intolerance manifesting as persistent nausea and vomiting. Tirzepatide was initiated at 2.5 mg weekly and titrated incrementally to 12.5 mg weekly over the three months preceding presentation. Her concurrent diabetes regimen included metformin, empagliflozin, and long-term insulin therapy. Following tirzepatide initiation, glycemic control improved markedly, with hemoglobin A1c declining from 8.1% to 6.6% (normal: below 5.7%) and BMI declining from 47.2 to 35.4 kg/m².

On presentation, vital signs were stable. Physical examination was notable for diffuse abdominal tenderness, abdominal distension, and diminished bowel sounds in the absence of peritoneal signs. Laboratory evaluation revealed no leukocytosis, lactic acidosis, or electrolyte disturbances to suggest bowel ischemia or perforation. Plain abdominal radiography demonstrated multiple small air-filled small bowel loops predominantly in the left upper quadrant, raising concern for ileus versus small bowel obstruction (Figure 1).

**Figure 1:**
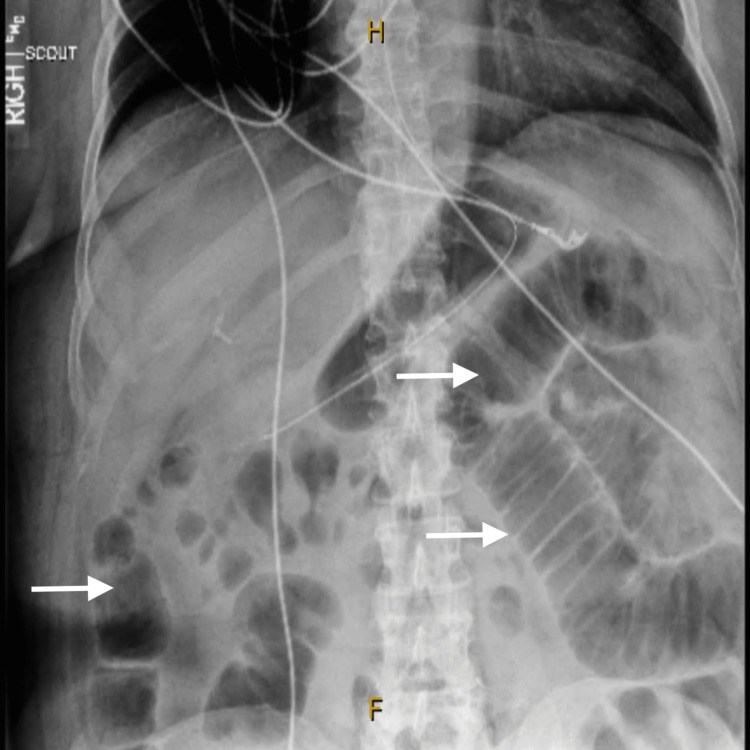
Abdominal imaging demonstrating an obstructive bowel gas pattern with multiple dilated small bowel loops and air-fluid levels (arrows), concerning for small bowel obstruction.

Subsequent small bowel follow-through confirmed obstruction. Computed tomography (CT) of the abdomen and pelvis demonstrated diffusely dilated small bowel loops extending to the distal ileum without evidence of perforation, bowel wall ischemia, obstructing mass lesion, volvulus, or a discernible transition point to suggest mechanical obstruction or malignancy (Figure 2).

**Figure 2:**
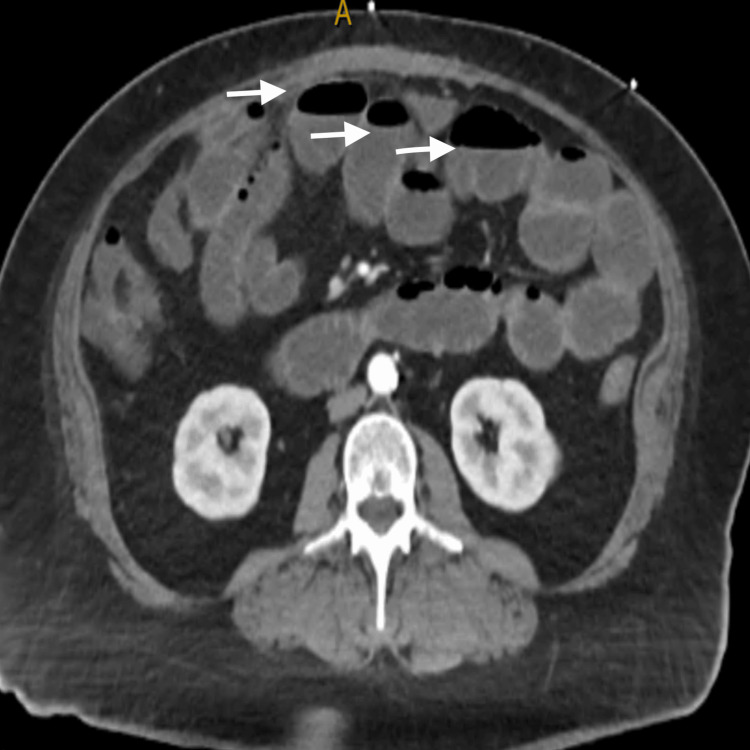
Computed tomography of the abdomen and pelvis demonstrating dilated fluid-filled small bowel loops with an obstructive bowel gas pattern (arrows), compatible with small bowel obstruction.

The patient was managed conservatively with nasogastric tube decompression, bowel rest, intravenous fluid resuscitation, and serial clinical monitoring. General surgery was consulted and recommended continued non-operative management given the absence of bowel compromise and the patient's prompt clinical improvement. The patient had no prior abdominal surgeries or known adhesions. A thorough evaluation for reversible etiologies, including electrolyte derangements, thyroid dysfunction, medication effects, and illicit substance use, was unrevealing. Among known predisposing conditions, longstanding diabetes mellitus with associated enteric neuropathy was identified as the sole underlying risk factor for dysmotility.

Given tirzepatide's established pharmacologic effects on gastrointestinal motility and the compelling temporal association between dose escalation and symptom onset, the medication was identified as the primary suspected contributing factor and was discontinued at discharge. The patient achieved complete symptomatic resolution and successfully advanced to a regular diet without recurrence of pain or emesis. Over two months of outpatient follow-up, she experienced no further episodes of small bowel obstruction.

## Discussion

Tirzepatide has rapidly emerged as a highly effective therapeutic agent for T2DM and obesity, with widespread adoption across diverse patient populations [1,2]. While gastrointestinal adverse effects are frequently encountered in clinical practice, severe motility disorders such as ileus and small bowel obstruction remain uncommon and incompletely characterized complications of incretin-based therapy.

The pathophysiology underlying tirzepatide-associated bowel obstruction is likely multifactorial. GLP-1 receptor activation delays gastric emptying, inhibits gastrointestinal smooth muscle contractility, and reduces small bowel transit through modulation of enteric neuronal pathways and vagal signaling [4,5]. These mechanisms collectively contribute to the "ileal brake" phenomenon, which physiologically slows intestinal transit and promotes satiety. Tirzepatide's dual GIP and GLP-1 receptor agonism may potentiate these inhibitory effects on gut motility beyond those observed with selective GLP-1 receptor agonists alone, potentially predisposing susceptible individuals to functional obstruction or severe ileus.

In the present case, several factors collectively implicate tirzepatide as the primary contributing etiology. The patient harbored multiple predisposing risk factors for gastrointestinal dysmotility, including longstanding T2DM, diabetic peripheral and autonomic neuropathy, morbid obesity, and a history of intolerance to multiple incretin-based therapies. Diabetic autonomic neuropathy is independently associated with impaired gastrointestinal motility, including delayed gastric emptying and intestinal transit, and may have amplified the motility-suppressing effects of tirzepatide in this patient [6]. Conversely, the absence of prior abdominal surgery, intra-abdominal adhesions, obstructing mass lesions, volvulus, or inflammatory pathology effectively excluded the more common mechanical etiologies of small bowel obstruction. The temporal association between dose escalation to 12.5 mg weekly and symptom onset, combined with the absence of an identifiable mechanical cause on cross-sectional imaging, is most consistent with a non-mechanical, functional obstruction precipitated by tirzepatide. Furthermore, rapid clinical improvement following conservative management and medication discontinuation, without surgical intervention or recurrence during follow-up, further strengthens this causal inference.

Notably, symptoms manifested following escalation to 12.5 mg weekly, raising the possibility of a dose-dependent relationship between tirzepatide and severe gastrointestinal dysmotility. The patient's prior intolerance to both semaglutide and dulaglutide may represent an important clinical signal, suggesting that a history of severe gastrointestinal adverse events with incretin-based therapies may identify patients at heightened risk for more serious motility-related complications with subsequent agents in this class.

These observations are consistent with an evolving body of pharmacovigilance and postmarketing surveillance data associating GLP-1 receptor agonists with serious gastrointestinal adverse events. Sodhi et al. reported significantly increased rates of bowel obstruction among patients using GLP-1 receptor agonists for weight loss compared with those receiving other weight management medications [7]. In 2023, the FDA updated semaglutide labeling to include ileus as a recognized adverse reaction following the accumulation of postmarketing reports. However, published evidence specifically implicating tirzepatide in bowel obstruction remains limited and is largely confined to rare case reports, highlighting the clinical importance of the present case amid the rapidly expanding use of tirzepatide for type 2 diabetes and obesity management [8].

An important diagnostic consideration highlighted by this case is that drug-induced functional bowel obstruction can closely mimic mechanical obstruction on clinical and radiologic grounds, potentially leading to unnecessary invasive intervention if the diagnosis is not recognized [9]. Clinicians should maintain a high index of suspicion for bowel obstruction in patients receiving tirzepatide or other incretin-based therapies who present with persistent abdominal pain, nausea, vomiting, distension, or constipation. Early recognition is essential, as delayed diagnosis carries the risk of bowel ischemia, perforation, and the need for surgical intervention. Particular caution is warranted in patients with diabetic autonomic neuropathy, preexisting gastroparesis, chronic constipation, or a prior history of gastrointestinal intolerance to GLP-1 receptor agonists.

As incretin-based therapies continue to expand in their indications and utilization, prospective studies and systematic postmarketing analyses are needed to better define the true incidence, underlying mechanisms, and patient-specific risk factors associated with severe gastrointestinal dysmotility and bowel obstruction. In the interim, this case advocates for individualized risk stratification and close gastrointestinal monitoring in high-risk patients initiated on tirzepatide therapy.

Further prospective research and systematic postmarketing surveillance are necessary to better define the true incidence, underlying pathophysiologic mechanisms, and patient-specific risk factors associated with tirzepatide-induced bowel obstruction, with the ultimate goal of informing safer prescribing practices and optimizing patient selection for incretin-based therapy.

## Conclusions

Tirzepatide (Mounjaro)-associated small bowel obstruction represents a rare but potentially serious adverse event that warrants clinical recognition as utilization of this medication continues to grow. This case illustrates that severe gastrointestinal dysmotility culminating in acute bowel obstruction can occur with tirzepatide therapy, particularly in patients with underlying diabetic autonomic neuropathy, longstanding enteric dysmotility, and prior intolerance to incretin-based agents - a constellation that may identify a uniquely vulnerable patient subset. Vigilance for this complication is especially warranted during periods of dose escalation, when motility-suppressing effects may be most pronounced. Prompt clinical recognition, timely discontinuation of the offending agent, and institution of conservative supportive measures can yield favorable outcomes and obviate the need for surgical intervention.
